# Coping Strategies Used by Older Cancer Survivors During the COVID-19 Pandemic: A Longitudinal Qualitative Study

**DOI:** 10.1177/01640275221120102

**Published:** 2022-09-01

**Authors:** Jacqueline Galica, Heather M. Kilgour, John L. Oliffe, Kristen R. Haase

**Affiliations:** 1 School of Nursing, Division of Cancer Care & Epidemiology, Queen’s Cancer Research Institute, 4257 Queen’s University, Kingston, ON, Canada; 2 School of Nursing, Faculty of Applied Science, 8166 The University of British Columbia, Vancouver, BC, Canada

**Keywords:** cancer, COVID-19, older adults, qualitative, coping skills, coping behaviors

## Abstract

**Objectives:** The objective of this study is to longitudinally examine the coping strategies used by older cancer survivors (≥60 years of age) during COVID-19. **Methods:** An interpretive descriptive approach was used to collect and analyse qualitative data collected via 1:1 telephone interviews at three timepoints: June/July 2020, January 2021, and March 2021. **Main Findings:** Coping strategies used by older adults reflected the resources available to them, and their agency in self-triaging and deciding on resources to support their coping. These decisions were impacted by pandemic-imposed restrictions and necessitated readjustment over time. Three themes were developed to describe coping strategies (including any changes): adapting means and methods to connect with others; being intentional about outlook; and taking actions toward a brighter future. **Conclusion:** Older adults used a variety of coping strategies, though their reliance on resources beyond themselves (e.g., family/friends) indicates a need to add tailored resources to existing professional services.

## Introduction

The coronavirus disease of 2019 (COVID-19) pandemic resulted in recurring and diverse disruptions around the globe. As the pandemic continued, there was a growing emphasis to understand the degree to which human lives were impacted (Chriscaden, 2020). These insights are useful to inform preparatory changes to mitigate negative implications of future pandemics (Morganstein, 2022). For instance, understanding how people coped during the COVID-19 pandemic will be useful, since some forms of coping (e.g., avoidance coping (Shamblaw et al., 2021)) may be maladaptive and result in psychological distress (Rettie & Daniels, 2020; Shamblaw et al., 2021) or intolerance of pandemic-related uncertainty (Rettie & Daniels, 2020). Other forms of coping are likewise important to distill (e.g., positive reframing, acceptance) since they contribute to improved mental health (Yıldırım et al., 2021) and resilience and recovery (Polizzi et al., 2020) in the face of the persistent and unpredictable nature of a pandemic. Furthermore, some sub-groups emerged as especially vulnerable during the COVID-19 pandemic, and such groups are important to study so that proactive social and economic policies can be enhanced and/or implemented for support during future pandemics.

Older adults were one such group who received specific attention (World Health Organization, 2021a; 2021b) during the COVID-19 pandemic (Rettie & Daniels, 2020). The greatest COVID-19 related stressors of older adults were confinement restrictions (Whitehead & Torossian, 2021), being concerned about others’ physical and mental health and safety (Whitehead & Torossian, 2021), as well as loneliness (Kobayashi et al., 2021; Whitehead & Torossian, 2021), the latter of which increased over the course of the pandemic (Van Tilburg et al., 2020). Greater COVID-19 related stressors were associated with poorer psychological wellbeing (Whitehead & Torossian, 2021), and to cope with these stresses, older adults used a variety of coping strategies wherein proactive strategies (e.g., preparing for an event (Pearman et al., 2021)) and social support (Fuller & Huseth-Zosel, 2021; Whitehead & Torossian, 2021) had the greatest impact on wellbeing. Indeed, a greater use of adaptive coping behaviours (e.g., seeking social support or existentially-related activities (Carver, 1997; Carver et al., 2010; Carver et al., 1989)) are believed to promote resilience that is critical for coping with ongoing stresses, such as those experienced during a pandemic (Polizzi et al., 2020).

Although older adults are an important population to address during a pandemic, those with chronic conditions, including cancer, may experience additional challenges that are potentially amplified by the ongoing nature of a pandemic. Recommendations for cancer care continuity during the COVID-19 pandemic were proposed (Desideri et al., 2020; Segelov et al., 2020); however, these did not address the unique period after cancer treatment was completed. Cancer survivorship for older adults is said to involve a period of adaptation, as they transition out of active cancer treatment and enter a new post-treatment period (Deimling et al., 2002; Fitch et al., 2021; Towsley et al., 2007). Indeed, older cancer survivors report a variety of challenges or needs (e.g., physical, emotional, nutritional, cognitive, etc. (Fitch et al.; Kleckner & Magnuson, 2022; Scotté et al., 2018; Watanabe et al., 2020)). Of these, physical challenges (e.g., physical capacity, symptoms/side effects, and changes in bodily function/appearance/falls (Fitch et al.; Sulicka et al., 2018; Watanabe et al., 2020) are most commonly experienced. These challenges are experienced across genders and cancer diagnoses indicating the importance of assisting all older adult cancer survivors as they transition out of cancer treatment, particularly during a pandemic when supportive resources may not be easily accessible.

### Conceptual Framework

In 2003, Moos and Holahan proposed an integrative conceptual framework useful to understand coping processes. Drawing from this framework, the coping strategies used by older adults during the COVID-19 pandemic (Fuller & Huseth-Zosel, 2021; Pearman et al., 2021; Whitehead & Torossian, 2021) can be broadly classified as stemming from their personal and/or environmental systems (Moos & Holahan, 2003). The personal system includes relatively stable personal characteristics, such as self-efficacy and general personality traits (Moos & Holahan, 2003). The environmental system, which is also said to be relatively stable, includes conditions in one’s environment, such as physical health, finances, and relationships with family and friends (Moos & Holahan, 2003). Both systems influence how individuals perceive and cope with life circumstances, such as a pandemic, which in-turn influences their overall health and wellbeing (Moos & Holahan, 2003).

### Rationale and Statement of Purpose

Research conducted early in the COVID-19 pandemic indicated that older adults draw from both their personal and environmental systems for coping (Galica et al., 2021); yet no known published research has examined how the coping strategies of older adult cancer survivors may change over the course of a pandemic. Considering that this understanding may be useful to inform preparatory changes to mitigate negative implications of future pandemics (Morganstein, 2022), the purpose of this article is to longitudinally examine the coping strategies used by older cancer survivors (≥60 years of age) during the COVID-19 pandemic. The specific research question is: What coping strategies do older cancer survivors use during the COVID-19 pandemic, and how do they change over time?

## Methods

We used an interpretive descriptive approach (Thorne, 2016), a qualitative methodology that permits researchers to choose from a range of qualitative traditions to integrate their disciplinary knowledge into the research process (Thorne, 2013; 2016; Thorne et al., 1997). Given the research team’s expertise in geriatric- and psychosocial-oncology, this approach was suitable for addressing the research question. A longitudinal research design was used to collect qualitative data at three timepoints over the course of the COVID-19 pandemic: June/July 2020, just as the first wave was ending; January 2021, at the peak of the second wave when vaccines began to be administered; and March 2021, as the third wave was starting (Canadian Institute for Health Information (CIHI), 2022).

### Recruitment and Sample

Using a homogeneous purposive sampling method, participants were recruited from a database of persons who had completed treatment for breast or colorectal cancer, been discharged from the (Cancer Centre of Southeastern Ontario) in (Kingston, Ontario, Canada) during the preceding 12 months, and indicated that they were agreeable to be contacted for subsequent research led by the first author. Persons in the database who were older adults (≥60 years of age) were invited to participate (Galica et al., 2021). Twenty-four participants engaged in the first interview, and 18 provided ongoing consent to complete the second and third interviews. Reasons for not participating in subsequent interviews were not formally collected although these individuals remained agreeable for us to use data from their first interview.

All participants consented for their clinical and treatment data to be extracted from the first author’s database. Ethical review for this study was obtained prior to data collection (Ethics file #6030148).

### Data Collection and Analysis

A semi-structured interview guide was used to collect qualitative data via 1:1 telephone interviews conducted by experienced qualitative researchers (JG and KH) and a graduate trainee (HK). Excerpts from the interview guides are included in Table 1; additional details have been published elsewhere (Galica et al., 2021; Kilgour et al., 2021). Prior to the third interview, we worked with a plain language editor to create a summary of findings to date. Given the limitations of member checking (Birt et al., 2016), we used the opportunity of the third interview to discuss the developing findings and probe further. The interviews lasted approximately 45-minutes, were audio-recorded, transcribed and checked for accuracy. After the data for all timepoints was collected, three authors (JG, HK and KH) read and re-read the transcripts of the initial interview and jotted brief notes to document preliminary descriptors and interpretations of the data. The second and third timepoint interview transcripts were similarly read paying particular attention to changes via constant comparative methods. The data collected for each timepoint were read and compared to distil patterns and explain variations, in accordance with interpretive description approaches. Two authors (JG and KH) met weekly to discuss and develop the coding structure to fracture the data. Driving consensus through conversations within the research team, the codes were organized into sub-themes and then subsumed into themes to conceptually describe the processes and discrete findings to address the research question. Illustrative quotes were used to demonstrate the fit of the data with the discrete findings. NVivo 12 software was used to house and code the data.

**Table 1.** Sample Questions from Interview Guides.

Time	Example interview questions
Interview 1 (June/July 2020)	Could you tell me what it’s like to be an older cancer survivor – someone who has completed treatment for cancer - in this pandemic?
	How do you think you’re doing with coping with your cancer-related concerns during the pandemic?
	Of the changes that you’ve gone through as an older cancer survivor during this pandemic, what stands out to you as the most important?
Interview 2 (January 2021)	In your first interview in the summer, we talked about cancer-related coping during the pandemic. I’d like to talk about changes you may have experienced since then.
	Have you had any major cancer related concerns since our last interview?
	Have there been challenges related to getting or taking medications or your ability to attend clinics?
	What are your thoughts about managing your cancer-related concerns going forward in the pandemic?
Interview 3 (March 2021)	Older adults were provided with key findings from interviews 1 and 2
	Older adults told us they were concerned that (a) their caregivers could not go with them to in-person appointments, (b) they had less support managing survivorship concerns, and (c) that their cancer might recur. They had mixed opinions about virtual appointments. Some liked not having to travel to in-person appointments in winter.
	We then asked reflective questions
	What do you think of these findings?
	Is there anything that doesn’t make sense to you?
	Which of these findings resonates most with you?

We used Thorne’s (2016) principles to ensure qualitative rigour throughout the analysis process. These include epistemological integrity (e.g., a longitudinal qualitative design consistent with the study purpose), credibility (e.g., codes and themes developed by two or more research team members), analytic logic (e.g., an audit trail to illuminate key decisions), and interpretive authority (e.g., use of exemplary quotations reflecting a range of participant perspectives).

### Findings

Participants were, on average, 71.1 years of age (*SD ±* 4.9), predominantly identified as female (*n =* 12, 66.7%) and Caucasian (*n =* 18, 100%). Most were married or common-law (*n =* 15, 83.3%) and had completed post-secondary education (*n =* 11, 61.1%) or higher. Eleven (61%) had been diagnosed with breast cancer, and the sample had completed treatment, on average, 19 months prior to first interview (*SD ±* 11). Full demographic and clinical characteristics are presented in Table 2.

**Table 2.** Demographic and Clinical Information.

Characteristic	Interview 1 (*n =* 24) *N* (%)	Interview 2 and 3 *(n =* 18) *N* (%)
Age (years)^a^	71.5 (63–83)	71.1 (63–80)
Gender		
Female	14 (58.3%)	12 (66.7%)
Male	10 (41.7%)	6 (33.3%)
Marital status		
Married or common-law	18 (75%)	15 (83.3%)
Widowed	2 (8.3%)	0 (0%)
Separated or divorced	1 (4.2%)	1 (5.6%)
Single (never married)	3 (12.5%)	2 (11.1%)
Education		
Up to high school graduate	5 (20.8%)	2 (11.1%)
Up to post-secondary graduate	13 (54.2%)	11 (61.1%)
Up to graduate-level graduate	6 (25%)	5 (27.8%)
Ethnicity		
Caucasian	22 (91.7%)	18 (100%)
Black	1 (4.2%)	0 (0%)
Indigenous	1 (4.2%)	0 (0%)
Cancer diagnosis		
Breast cancer	12 (50%)	11 (61.1%)
Colon or rectal cancer	12 (50%)	7 (38.9%)
Cancer treatment		
Radiation	13 (54.1%)	13 (72.2%)
Chemotherapy	23 (95.8%)	17 (94.4%)
Other^b^	6 (25%)	6 (33.3%)
Time since treatment (months)^a^	19.4 (1–41)	19.4 (1–41)

^a^Mean (range).

^b^As indicated by participants (e.g., immunotherapy, surgery, injections).

The coping strategies used by older adults during the COVID-19 pandemic reflected the resources available to them, and their agency in contemplating choices and deciding on resources to support their coping. These choices were often impacted by restrictions imposed by the pandemic and therefore necessitated adjustment as the waves of the pandemic unfolded. We organized the coping strategies used by older adults, and changes in the strategies they used over the pandemic, into three themes: adapting means and methods to connect with others; being intentional about outlook; and taking actions toward a brighter future.

### Adapting Means and Methods to Connect With Others

Participants described how they modified the means and methods by which they connected with others over the course of the pandemic. Within this context older adults’ support networks – relationships with family, friends, neighbours, healthcare providers and pets – had an important impact on their coping during the pandemic. These networks supported coping in the form of instrumental (e.g., grocery delivery) and emotional (e.g., motivation, reassurance) supports. However, as the pandemic persisted, the means and methods of connecting with these supports shifted.

Although the benefits of having a large support network were apparent, participants with small networks spoke of their unique challenges, such as living alone without family support nearby. One participant described: “*I’m terrified of getting this COVID disease, because I live alone. Everybody that lives alone, I think is a little more concerned about the pandemic than people that have a big family to back up, (to) rely on*” (P08). This pervasive concern led these individuals to regularly check in on friends and neighbours to ensure their needs were being met (e.g., telephone calls every few days). In this way, those without family members living nearby relied on neighbours for instrumental support although emotional support was received from family members who lived farther away (e.g., across Canada or internationally) by using video conferencing platforms or telephone. One participant described the positive impact of seeing her extended family members for short outside and physically distanced visits. She said: “*One bright spot for us, is a daughter and her family [who live] 40* *minutes away so we do some outdoor visits with them. … Something like that, it carries you along for a while*” (P23).

Having the means to live in the country and own private land was an advantage as participants described enjoying gardening, cross-country skiing, or inviting family members to camp as means to cope with the uncertainty of the pandemic. One participant spoke of her child and grandchildren setting up their RV in her backyard so that everyone had their own space and washroom and could physically distance outside during the day. Other participants described the isolation on their private acreage left them without fear of “*catching anything [COVID-19]*” (P03). In this way, the focus on space and place indicated rural advantages for some participants wherein they could adapt to physical distancing restrictions to safely accommodate visits with extended family. Furthermore, the idea of being locked down was eased with the area and remoteness of their properties in which they lived. However, as the pandemic wore on, geographic aspects that supported initial coping during the pandemic in the form of physical distancing had varying impacts for ongoing coping. One participant, who self-identified as a recluse all of her life, began to wonder: *“[am I] getting a little depressed because I do spend so much time alone. I used to love it, but I find as I get older, I wish somebody would drop in a little more often. I don’t know if it’s depression [or] the winter blues*” (P08). Interestingly, this statement reflects the limits of adapting during the pandemic in that lock downs enforced individuals living alone to remain alone, rather than permitting them freedom to make this lifestyle choice.

Participants described a *pandemic fatigue* whereby the camaraderie that was prevalent among their networks at the beginning of the pandemic diminished as time wore on. One participant described: “*we have a very tight knit street area. … although, things are changing; we have lost that camaraderie that we did every Friday night [last summer]*” (P13). Aware of the uncertainty of the pandemic’s duration, participants brainstormed with their networks about new/alternative strategies for connecting, which included Zoom calls, or pooling money for birthday gifts for a single drop off.

In the cancer context, participants generally accepted the continued use of technology in the form of virtual health care appointments. One participant described their perspective about in-person and virtual visits: “*I suspect going into the future we will have a mix of those two again. I think there will be an initial assessment over the computer and then, if necessary, in-person. Certainly the hours and hours of sitting waiting to get into appointments would be reduced at this point. … especially the elders, the ones with less mobility, where everything’s such an effort to get out, that the virtual would be very helpful*” (P23). In this regard, adapting also extended into the cancer context whereby participants initially lacked confidence with having virtual consultations. However, as time went on, their confidence with using those technologies and acceptance that virtual appointments afforded them safety (from exposures to COVID) knitted together to normalize that change and virtual connectedness. Moreover, such change was understood and anticipated as continuing after COVID-19.

### Being Intentional About Outlook

Regardless of the quantity and quality of coping resources available to them, participants spoke of being intentional about outlook, and their thoughts and management of influencing factors. Many participants described having a positive or optimistic outlook as a source for coping with the ongoing pandemic. One participant put it simply: “*you choose how you think. Don’t worry about things because it doesn’t help one bit*” (P02). Another participant described how this perspective had evolved over the pandemic saying: “*I was always thinking about the worst at the start [of the pandemic] and then I kind of got to the point where … I realized that worry isn’t going to change anything on it, so you might as well do what you want to do and accomplish what you can*” (P06). Herein, some participants were frozen early on by the pandemic having lost routine and purpose in the everyday. However, as time wore on, there was renewal of ‘doing’ and being ‘busy’ within the limits of COVID to reinstate some semblance of control and normalcy.

Participants used technology to seek out information about the status of the pandemic and public health guidelines. But as the pandemic wore on, participants described feeling fed up with the relentless and often conflicting media reports about pandemic-related matters, such as rising death tolls, repeated and unmet political promises for more vaccines, and peoples disregard for public health recommendations. One person stated, “*it’s frustrating, because there’s a lot of people that aren’t taking it all that serious – I think it’s kind of dragging it out*”, while another believed that “*the longer it [the pandemic] goes on the more people get more lackadaisical about it, and that’s why they’re having more outbreaks*” (P06). In this way, participants expressed frustration whereby they wanted the pandemic to “*go away*” (P15) so that they could resume their activities, such as getting groceries, and not have to worry or be fearful of catching COVID-19. However, the ongoing reports of variants of concern and vaccine effectiveness were identified by one participant who stated: “*I’m getting sick, or tired, or something of hearing constantly the news that only gets worse, and worse, and worse. And then the next thing you know now you’ve got all these variants. Now you don’t have enough vaccines. And now you’re on the low end of the list for the vaccines. It’s like ‘oh no’*” (P12). In this way, participants described having to repeatedly and intentionally revise their expected timelines about the pandemic’s end and anticipated future pandemics.

Nevertheless, participants rationalized their ongoing adherence to public health recommendations as small compromises in their life as opposed to the rising COVID-related death rates. One participant said “*I can accept what we have to do. … I mean, we’re not being asked to go to war*” (P13). In this manner, participants perceived their individual responsibilities relative to a global health emergency. Although they understood the reasons for not being able to see each other in person, participants looked forward to opportunities to physically meet with members of their support networks. One participant described discussions with her support networks as “*we’re all saying the same thing: ‘when this is over, we’re going to have one great big party and get together’*” (P28). In this way, these forward-looking opportunities – like visualization of a post-pandemic return and celebration to mark that end - were important as older adult cancer survivors coped with the ongoing uncertainty of the pandemic.

However, the positive outlook that participants described seemed to be related to their ongoing good health and reassurance that cancer had not recurred. Indeed, fear of cancer recurrence continued to be an ongoing concern for participants, one of whom described it as their “*biggest fear*” (P07). To help cope with these fears, participants relied on routine screening for recurrence that their health care team monitored. The impact of receiving a negative finding for recurrence (e.g., CT scan and colonoscopy) was apparent, as expressed by one participant: *“[it] improves your attitude. It takes some of the worry away from being a cancer survivor*” (P18). However, despite two years of being in remission, another participant described “*if it [cancer] did pop up, I don’t know what I’d do*” (P10). Participants expressed concern about needing to access health resources in public settings (e.g., hospitals or clinics), which participants had contrasting views about. For instance, one participant was “*happy to go there [cancer follow-up clinic] and ask some questions*” (P12) whereas another did “*not want to go anywhere near a hospital or a clinic or a cancer centre*” (P10). Both perspectives reflect choices wherein participants had to be intentional in their outlook to pursue cancer and/or health care as needed. This self-triaging - in many respects – enabled older cancer survivors to be agentic in pursuing virtual or in person care, or any combination thereof. This choice may have been helpful to frame older adults’ outlook in that it provided some element of control in an otherwise uncertain pandemic and the ever-present fear of cancer recurrence. Nevertheless, some participants explained that they don’t dwell on the possibility of cancer recurring and take every day as it comes, even while coping with ongoing sequelae of cancer (e.g., having multiple bowel movements as an outcome of cancer and its treatments). Indeed, it seemed as though the combined experiences of having cancer and living during the pandemic provided new insights for coping during the pandemic. One participant described this as: *“…things like that we’ve done, that maybe if I hadn’t been sick and if it hadn’t been COVID*” (P13).

### Taking Actions Toward a Brighter Future

In addition to being intentional in their thoughts and outlook, participants consistently described taking actions toward a brighter future. For instance, participants spoke about their leisure time during retirement wherein they regularly travelled, but due to the pandemic, their travel plans were revised or cancelled. Although they recognized that post-pandemic travel would be different, they looked forward to resuming such travel before experiencing health challenges due to their advancing age and cancer. One participant stated: “*I love to be active and I love to travel and I want to be able to do all those things.…. I’ve had two dear friends that have been diagnosed with cancer in the last year and it’s like, ‘oh, geez’, I’m at that age when this is going to happen*” (P28). Because of pandemic-imposed restrictions at recreational facilities where they could maintain their physical health (e.g., for golf and tennis), participants found alternatives, such as daily walks. However, as the 2020 Canadian winter approached, these efforts to maintain physical health dwindled and participants found themselves engaging in new activities. One participant described: “*never would I have ever imagined that literally I would get up, have breakfast, shower and then sit on the couch and watch TV all day. … if it was summer - even if it was lockdown - we would be outside*” (P13). This change illuminates the challenge of maintaining pre-pandemic activities as the COVID-19 pandemic wore on. However, to cope with the pandemic and promote their wellbeing, participants described a variety of activities that they intentionally pursued. One participant regarded these activities as a way to stay as healthy as possible and reduce pressure on health systems. To do this, participants prioritized their self-care, which one participant described as: “*doing what you have to do to make sure that you stay healthy during this [the pandemic]*” (P25). Participants cited a number of activities to promote their health and wellbeing, such as reading, board games, knitting, colouring, online fitness classes, and restoring cars. They also limited the number of hours that they watched TV and news, which seemingly permitted them with some sense of control over the amount time that COVID-19 impacted their daily lives. Their wellbeing was also supported by maintaining their social life (e.g., via telephone) and the enjoyment they received from engaging in hobbies that distracted them from the pandemic. One participant stated: “*I have a lot of ability to fix things or do stuff and nothing I love better than using my hands and having to think about what I’m doing. … Keep the mind active”* (P18). Participants spoke of the importance of developing a daily or weekly routine so as to provide some structure to their lives. One participant described: “*I need to make an action plan every Monday, so that I do something, because it’s too easy to do nothing. But if you plan something, you have to do it. So that’s helped tremendously*” (P12). Other participants described the importance of keeping a daily routine or making lists to prompt them to complete tasks each day.

With the advent of vaccines, participants described a sense of relief and urgency about when they were going to get their first dose. One participant expressed this urgency stating: “*will the vaccinations hurry up so I can be sure that I won’t get COVID or I have less of a risk*” (P25). In this way, participants were aware of still being susceptible to COVID-19 post-vaccination but believed that “*the vaccine makes a big difference*” (P13). Receiving the COVID-19 vaccination appeared to represent a step closer toward the pandemic’s end, where older adults could resume activities that they formerly enjoyed. One participant described her hopes after being vaccinated: “*I think that will certainly open the door to us being able to connect again, because most of our friends are all in the same boat that we are*” (P09).

Participants recognized that there would be a “*new normal*” post-pandemic, where life was “*not going to be the same as it ever was*” (P10). Participants described this new normal as ongoing restrictions in public places (e.g., hospitals or restaurants), wearing masks in communal areas, and staying home if they felt even the slightest bit unwell. But this new normal wasn’t seen as problematic as expressed by one participant: “*I don’t think we’ll ever get back to what I knew as normal. I mean just like after 9/11, travel changed then. … It’s not a bad change. It’s just a change. And I think we will see changes after COVID*” (P13). In this way, participants recognized that the ordinary everyday as they knew it was forever changed. However, they looked forward to the loosening of current restrictions. One participant described this hope: “*Even though it may be a new normal just being able to go out and do things or go places or visit people without having to be paranoid. … That’s what I look forward to, being able to have freer movement without having to worry about whether you’re going to get sick or get somebody else sick*” (P18).

## Discussion

This longitudinal qualitative study examined the coping strategies used by older adult cancer survivors during the COVID-19 pandemic including changes in the strategies that they used. Participants described how they adapted the means and methods by which they connected with people in their support networks and how they were intentional in their outlook during and beyond the pandemic. They expected post-pandemic life to be different but took actions toward brightening their futures as much as possible, regardless of their present or future reality. In what follows, we discuss our findings in relation to Moos and Holahan’s (2003) systems framework for coping and bridge to implications for social and cancer care for older adults. This integrative approach to applying the findings is especially important in preparing for future challenges including another (Witham et al., 2021) or unending pandemic.

The individual activities (e.g., gardening, restoring vehicles, etc.) used by older adults in this study are consistent with those identified in other research during the COVID-19 pandemic (Fuller & Huseth-Zosel, 2021; Galica et al., 2021). These activities reflect coping strategies of the personal system, as described by Moos and Holahan (2003), from which older adults drew more heavily during the pandemic as compared to their environmental systems (Galica et al., 2021). Indeed, participants’ need to draw from their personal systems may have been necessitated by lockdowns and isolation requirements. However, it is notable that the system – either personal or environmental (Moos & Holahan, 2003) - from which older adults predominantly drew their coping strategies fluctuated as the COVID-19 pandemic went on. As the pandemic persisted, participants used alternative methods for connecting with their family and/or friends for emotional and/or instrumental support, which may reflect the limitations of personal systems during a pandemic, or the inclination of older adults to ‘dig deeper’ and draw from their environmental systems as the need for coping supports continued. These adaptations reflect the importance that older adults placed upon their environmental systems for coping and therefore prompted them to use strategies they had never used (e.g., online conferencing software, camping and visiting in yards). Over time, however, the environmental system for coping seemed to fatigue (e.g., lost camaraderie of neighbours) requiring older adults to again turn inward and rely on their personal systems (e.g., intentional outlook, activities to support wellbeing). Nevertheless, older adults still looked for ways to connect with their environmental systems (e.g., using technology to watch media and connect with others) although this may have added to the fatigue that their personal systems were experiencing (e.g., desire for in-person connection). In this way, the environmental system seemed especially imperative to support the coping of older adults, so much so that they made changes to their personal systems (e.g., decisions to vaccinate, adherence to public health guidelines, engaging in activities to stay healthy) in order to expedite their ability to tap into their environmental systems again (e.g., in-person connections, travel, and other recreational activities). Overall, study findings reflect a reciprocal interaction of older adults’ personal and environmental systems (Moos & Holahan, 2003) to support their coping, although their environmental systems seemed to be highly influential over the long-term. Indeed, this premise has been supported wherein people who prioritize pro-social and communal interactions to promote unity and fight divisiveness supported their coping with COVID-19 related challenges (Kruglanski et al., 2021).

### Clinical Implications

Study findings and Moos and Holahan’s (2003) systems framework for coping are both useful to inform the clinical implications stemming from this work. Findings in this and other studies identify the relationship between using technology as a resource to support older adults’ coping during the pandemic. We find that technology was both helpful (e.g., as an environmental system strategy to connect with support networks and attend online fitness classes) and unhelpful (e.g., as a personal system strategy when learning about death tolls or about delayed vaccination roll-outs) for participants in this study, which is consistent with other literature (Garfin, 2020). For instance, technology (e.g., virtual reality fitness (Gao et al., 2020)) improved the physical outcomes of older adults (e.g., enhanced motor ability, reduced obesity) as well as their cognition and psychological outcomes (Gao et al., 2020). Similarly, reduced exposure to news/updates about COVID-19 was used as a coping strategy (Ogueji et al., 2021) and associated with fewer anxiety and depression symptoms (Fullana et al., 2020), and our participants described limiting the time spent watching daily news as helpful to support their coping. Other seemingly ‘simple’ coping strategies were used by participants (e.g., engaging in hobbies, developing a routine) to protect against anxiety and depressive symptoms during the COVID-19 pandemic and lockdown (Fullana et al., 2020). Indeed, the unique adaptive mechanisms used by older adults have been proposed as reasons for their wellbeing during the COVID-19 pandemic (Minahan et al., 2021) and may be reason for their fewer mental health complaints in the later stages of the pandemic (Fancourt et al., 2021).

These collective findings have implications for clinicians caring for older adults in the wake of the current and in anticipation of future pandemics wherein public health guidelines limit social and service provider interactions. For instance, technological resources can be availed as a source to maintain connection among older adults and their environmental systems (e.g., social networks); however older adults should be reminded of the limitations to such connections on their personal system (e.g., watching too much news). To support older adults in this regard, clinicians can counsel these individuals to inventory and access resources within their environmental systems and/or to engage in individual activities and/or hobbies that they enjoy within their personal systems.

### Strengths and Limitations

A strength of this study is the longitudinal design and distillation of shifting perspectives across the COVID-19 pandemic from a diverse sample (e.g., genders and rural/urban locations) comprising older adults who are cancer survivors. In terms of limitations, an additional time point or two for data collection, given the ongoing nature of the COVID-19 pandemic and the anticipated challenges with transitioning thereafter, would have strengthened the current study. That said, collecting data at multiple timepoints may have resulted in recall bias, for which additional data collection points may have also been impacted. Furthermore, we did not collect information about participants’ performance or functional status, which may influence the interpretation and applicability of results, particularly in relation to participants’ outlooks and actions taken toward a brighter future.

## Conclusion

Older adult cancer survivors use a variety of coping strategies that stem from both their personal and environmental systems (Moos & Holahan, 2003). However, environmental systems, such as relationships with family and friends, seem key to supporting their long-term coping during the COVID-19 pandemic. These findings can be useful to develop online infrastructure and support for older adults to ensure their connectedness to these important sources of emotional support during periods when in-person connection is not possible (e.g., an unending or future pandemic). In addition, these findings underscore the importance for community-based services to adapt and/or sustain online programming for older adults, particularly to prevent any isolating effects routinely experienced with increasingly volatile weather changes (e.g., extreme heat or cold).
